# Supplementary material for: ProteinShader: illustrative rendering of macromolecules
Source: BMC Struct Biol. 2009 Mar 30;9:19. doi: 10.1186/1472-6807-9-19 (PMC2672931; doi:10.1186/1472-6807-9-19)
Supplement: Additional file 1 — ProteinShader program without source code. This compressed file contains the complete ProteinShader program including associated libraries, but no source code. A README.txt file gives an overview of the ProteinShader distribution, and the index.html file in the help subdirectory has directions on getting started with the program as well as a set of tutorials. [file 1472-6807-9-19-S1.zip › ProteinShader-beta-0_9_4-binary/help/api/org/proteinshader/graphics/exceptions/class-use/GlyphException.html]

Uses of Class org.proteinshader.graphics.exceptions.GlyphException (ProteinShader API)


|  |  |  |
| --- | --- | --- |
| **Overview** \| **Package** \| **Class** \| **Use** \| **Tree** \| **Deprecated** \| **Index** \| **Help** | |  |
| PREV   NEXT | **FRAMES**    **NO FRAMES**     **All Classes** |  |


---


## **Uses of Class org.proteinshader.graphics.exceptions.GlyphException**

| Packages that use GlyphException | |
| --- | --- |
| **org.proteinshader.graphics.adapter** | Holds the StructureToGraphics class, which is used to manage the use of the drawing classes and OpenGL display lists. |
| **org.proteinshader.graphics.exceptions** | Holds the exception classes needed by org.proteinshader.graphics and its subpackages. |
| **org.proteinshader.graphics.typography** | Holds the classes needed for applying text labels to the curved surfaces of tubes and ribbons. |

Uses of GlyphException in org.proteinshader.graphics.adapter

| Methods in org.proteinshader.graphics.adapter that throw GlyphException | |
| --- | --- |
| `void` | `StructureToGraphics.readGlyphSet()`             Obtains a set of glyphs by using the default '.conf' configuration file in the fonts directory. |

Uses of GlyphException in org.proteinshader.graphics.exceptions

| Subclasses of GlyphException in org.proteinshader.graphics.exceptions | |
| --- | --- |
| `class` | `GlyphConfigException`             To report errors that occur while creating a Glyph. |
| `class` | `GlyphImageException`             To report errors that occur while creating a Glyph. |

Uses of GlyphException in org.proteinshader.graphics.typography

| Methods in org.proteinshader.graphics.typography that throw GlyphException | |
| --- | --- |
| `void` | `TextLabelFactory.readGlyphSet()`             Obtains a set of glyphs by using the default '.conf' configuration file in the fonts directory. |
| `void` | `TextLabelManager.readGlyphSet()`             Obtains a set of glyphs by using the default '.conf' configuration file in the fonts directory. |
| `GlyphSet` | `GlyphSetFactory.readGlyphSet(Properties p)`             Reads information from a source and constructs a GlyphSet object. |
| `GlyphSet` | `GLF2GlyphSetFactory.readGlyphSet(Properties p)`             Reads information from a '.glf' file and constructs a GlyphSet object. |
| `void` | `TextLabelFactory.readGlyphSet(String config)`             Reads the config file given as an argument and use the info to create a GlyphSet. |

---


|  |  |  |
| --- | --- | --- |
| **Overview** \| **Package** \| **Class** \| **Use** \| **Tree** \| **Deprecated** \| **Index** \| **Help** | |  |
| PREV   NEXT | **FRAMES**    **NO FRAMES**     **All Classes** |  |


---

# *Copyright © 2007-2008*
